# Post-traumatic stress disorder and the risk of violent crime conviction in Sweden: a nationwide, register-based cohort study

**DOI:** 10.1016/S2468-2667(23)00075-0

**Published:** 2023-06-01

**Authors:** Anabelle Paulino, Ralf Kuja-Halkola, Seena Fazel, Amir Sariaslan, Ebba Du Rietz, Paul Lichtenstein, Isabell Brikell

**Affiliations:** Department of Medical Epidemiology and Biostatistics, Karolinska Institutet, Stockholm, Sweden; Department of Psychiatry, University of Oxford, Oxford, UK; Department of Medical Epidemiology and Biostatistics, Karolinska Institutet, Stockholm, Sweden; Department of Psychiatry, University of Oxford, Oxford, UK; Department of Psychiatry, University of Oxford, Oxford, UK; Department of Medical Epidemiology and Biostatistics, Karolinska Institutet, Stockholm, Sweden; Department of Medical Epidemiology and Biostatistics, Karolinska Institutet, Stockholm, Sweden; Department of Medical Epidemiology and Biostatistics, Karolinska Institutet, Stockholm, Sweden

## Abstract

**Background:**

Post-traumatic stress disorder (PTSD) has been linked to violent crime in veteran populations. However, whether there is a link between PTSD and violent crime in the general population is not known. This study aimed to investigate the hypothesised association between PTSD and violent crime in the Swedish general population and to investigate the extent to which familial factors might explain this association using unaffected sibling control individuals.

**Methods:**

This nationwide, register-based cohort study assessed individuals born in Sweden in 1958–93 for eligibility for inclusion. Individuals who died or emigrated before their 15th birthday, were adopted, were twins, or whose biological parents could not be identified were excluded. Participants were identified and included from the National Patient Register (1973–2013), the Multi-Generation Register (1932–2013), the Total Population Register (1947–2013), and the National Crime Register (1973–2013). Participants with PTSD were matched (1:10) with randomly selected control individuals from the population without PTSD by birth year, sex, and county of residence in the year of PTSD diagnosis for the matched individual. Each participant was followed up from the date of matching (ie, the index person’s first PTSD diagnosis) until violent crime conviction or until being censored at emigration, death, or Dec 31, 2013, whichever occurred first. Stratified Cox regressions were used to estimate the hazard ratio of time to violent crime conviction ascertained from national registers in individuals with PTSD compared with control individuals. To account for familial confounding, sibling analyses were conducted, comparing the risk of violent crime in a subsample of individuals with PTSD with their unaffected full biological siblings.

**Findings:**

Of 3 890 765 eligible individuals, 13 119 had a PTSD diagnosis (9856 [75·1%] of whom were female and 3263 [24·9%] of whom were male), were matched with 131 190 individuals who did not, and were included in the matched cohort. 9114 individuals with PTSD and 14 613 full biological siblings without PTSD were also included in the sibling cohort. In the sibling cohort, 6956 (76·3%) of 9114 participants were female and 2158 (23·7%) were male. Cumulative incidence of violent crime convictions after 5 years was 5·0% (95% CI 4·6–5·5) in individuals diagnosed with PTSD versus 0·7% (0·6–0·7) in individuals without PTSD. At the end of follow-up (median follow-up time 4·2 years, IQR 2·0–7·6), cumulative incidence was 13·5% (11·3–16·6) versus 2·3% (1·9–2·6). Individuals with PTSD had a significantly higher risk of violent crime than the matched control population in the fully-adjusted model (hazard ratio [HR] 6·4, 95% CI 5·7–7·2). In the sibling cohort, the risk of violent crime was also significantly higher in the siblings with PTSD (3·2, 2·6–4·0).

**Interpretation:**

PTSD was associated with increased risk of violent crime conviction, even after controlling for familial effects shared by siblings and in the absence of SUD or a history of violent crime. Although our results might not be generalisable to less severe or undetected PTSD, our study could inform interventions that aim to reduce violent crime in this vulnerable population.

**Funding:**

None.

## Introduction

Post-traumatic stress disorder (PTSD) is reported to be prevalent in 3·9% of the global population^1^ and is characterised by exposure to a stressful event or situation, followed by persistent, intrusive recall of the stressor and avoidance of stressor-related circumstances.^2^ One of the criteria for PTSD diagnosis is “marked alterations in arousal and reactivity associated with the traumatic event(s)”,^3^ which can be indicated, in part, through “irritable behavior … typically expressed as verbal or physical aggression towards people or objects”.^3^ Because of these diagnostic criteria, previous research^4,5^ has hypothesised and shown that PTSD is linked to aggression and increased risk of violent behaviour in military veterans. One hypothesis for this link is that PTSD leads to dysregulation in stress-mediating biological systems in response to trauma,^6^ which leads to a fight-or-flight reaction to stress. Therefore, individuals with PTSD might have increased liability for aggression.

Research in context **Evidence before this study**

We searched PubMed for articles on the association between post-traumatic stress disorder (PTSD) and violence using the search terms “PTSD” or “post-traumatic stress disorder” or “trauma” and “violence” or “violent crime”. Our search included results from database inception to Dec 31, 2021, with no language restrictions. We identified two meta-analyses associating PTSD symptoms with intimate partner violence and increased likelihood of criminal justice involvement, suggesting an association between PTSD and violent crime. Available research has primarily focused on this association in military veterans, and most previous studies have used a cross-sectional design or relied on small sample sizes or specific populations, so generalising these findings is difficult.

We did not identify any studies evaluating the degree to which an association between PTSD and violent crime could be due to shared risk factors, such as familial factors, or of the role of confounding in the association of PTSD on violent crime. Consequently, there is little knowledge about the association between PTSD and violent crime in the general population.

**Added value of this study**

Using national register data from Sweden, this study provides a large and representative dataset of the Swedish population with up to 27 years of follow-up. Throughout follow-up, individuals with a PTSD diagnosis had higher absolute risk of being convicted for violent crime than individuals with no PTSD diagnosis. In the Cox regression, individuals with PTSD were at higher risk of committing a violent crime than control individuals without a PTSD diagnosis matched on birth year, sex, and county of residence in the year of diagnosis. Through comparing the hazards in differentially exposed siblings, we were able to adjust for unmeasured familial confounding factors. Individuals with PTSD had substantially increased risk for violent crime conviction compared with both control individuals in the general population and their full siblings without PTSD. This risk remained significant even after adjusting for parental characteristics, psychiatric comorbidities, and history of violent crime.

**Implications of all the available evidence**

PTSD, especially in combination with a history of violent criminality and substance use disorder, should be considered a risk factor for violent criminality and taken into account when developing crime reduction strategies.

In the non-military general population, other traumas, including physical and sexual assault, robbery, bereavement, political unrest, and natural disasters, have been linked to increased risk of PTSD.^7,8^ A nationwide study published in 2022 showed that being female, older age, having a lower education level, lower household income, living in an urban area, single parenthood, and immigrating from conflict-affected areas were associated with risk of PTSD in Sweden (a country without war experience).^9^ Whether there is a link between the development of PTSD and subsequent perpetration of violence in the general population, however, is unclear. Furthermore, the extent to which such an association between PTSD and violence is due to a causal effect is not known.

The association between PTSD and violent crime has, thus far, primarily been studied in military veterans. Meta-analyses have shown that PTSD symptoms among military veterans are significantly associated with domestic partner violence^10^ and violent-crime arrests.^11^ Among people who are incarcerated worldwide, PTSD prevalence is between 4% and 32% in men and between 16% and 58% in women.^12^ The association might also be present in the global population,^10,11^ as PTSD has been associated with perpetrating interpersonal violence^13^ and increased likelihood of incarceration^14^ in civilians. Although these studies indicate an association between PTSD and violent crime, the current literature has several limitations. Most studies have been cross-sectional^15^ with relatively small samples^14^ or non-representative subsets^16^ of the population. Furthermore, most studies have relied on self-reported symptoms, which are subject to recall bias, and results might not extend to populations with clinically diagnosed PTSD. These limitations can be addressed by studying the association of register-based, clinically diagnosed PTSD and violent-crime convictions in large representative population samples with longitudinal follow-up.

The mechanisms linking PTSD to increased risk of violence perpetration are not well understood. This link might be confounded by environmental factors (eg, socioeconomic status) or other psychiatric disorders previously associated with PTSD and violent crime (eg, depression, substance use disorder [SUD], psychotic disorders, or behavioural disorders).^17^ Familial risk factors, including genetic and environmental influences, might also confound the association between PTSD and violent crime. These familial factors are important to consider as previous studies have reported that PTSD^18^ and violent crime^19^ tend to aggregate in families and are heritable, with twin studies estimating heritability of around 30–50%^18^ for PTSD and 50–60%^20^ for violent crime. Sibling-comparison studies provide one way to adjust for potential unmeasured familial confounding,^21^ and have previously been used to study the association between multiple psychiatric disorders—including schizophrenia, bipolar disorder, depression, and attention-deficit hyperactivity disorder (ADHD)—and violent crime.^16^ However, to our knowledge, no sibling studies have investigated the link between PTSD and violent crime.

The aims of this study were to investigate the hypothesised association between PTSD and violent crime in the general population and to investigate the extent to which familial factors might explain this association with national register data from Sweden. Furthermore, as comorbid PTSD and substance abuse are common in criminal settings, we investigated the risk for violent convictions in individuals with different violence and SUD histories.

## Methods

### Study design and data sources

In this nationwide, register-based cohort study, we used personal identification numbers assigned to everyone born or registered in Sweden^22^ to link multiple longitudinal registers. We identified data for inclusion between Dec 15, 2021, and Feb 15, 2023 and all data were input into software for analyses by Feb 15, 2023. This study was approved by the regional ethics review board in Stockholm, Sweden (2013/862-31/5).

Participants were included from the National Patient Register, the Multi-Generation Register, the Total Population Register, and the National Crime Register. The Total Population Register contains date-of-birth information for everyone residing in Sweden since 1947.^22^ The Multi-Generation Register connects all individuals born in Sweden since 1932 and registered as alive and living in Sweden on Jan 1, 1961, to their family members.^23^ The National Patient Register provides psychiatric inpatient hospitalisation data since 1973 and outpatient specialist care visits since 2001, coded with the International Classification of Diseases (ICD) eighth edition (ICD-8), ninth edition (ICD-9), and tenth edition (ICD-10).^24^ The National Crime Register contains information for all convictions in Swedish general courts since 1973,^19^ with almost complete national coverage.^21^ In Sweden, individuals found guilty of committing a violent crime are convicted regardless of their mental health status,^19^ allowing for the investigation of PTSD in relation to violent-crime convictions. The age of criminal responsibility is 15 years in Sweden; therefore, no crimes committed before age 15 years are registered. Linked register data were available until 2013.

### Study population

All individuals born in Sweden between 1958 and 1993 were assessed for eligibility for inclusion in this study. We excluded individuals who died or emigrated before their 15th birthday, were adopted, were twins, or whose biological parents could not be identified (appendix p 13). We excluded twins from this study as monozygotic twins share more genetics than full siblings, potentially biasing the within-sibling estimates and making their inclusion in sibling-control analyses unsuitable. Furthermore, there was insufficient statistical power to conduct separate sibling-control analyses on twins only.

From this population, we identified all individuals diagnosed with PTSD, defined in the National Patient Register by an inpatient or outpatient visit with a primary or secondary diagnosis of PTSD under ICD-9 code 309B or ICD-10 code F43.1. The validity of PTSD diagnoses in the National Patient Register has been shown to be sufficient for epidemiological research, with a positive predictive value of 84%.^25^ We then matched each individual with PTSD on sex, birth year, and county of residence in the year of diagnosis to ten individuals from the general population who were alive, living in Sweden, and without a PTSD diagnosis at the date of matching (ie, the date of the index person’s first PTSD diagnosis in the National Patient Register). The county of residence for matched control individuals was defined as the county of residence at the year of diagnosis for the index person with PTSD they were matched with.

If matched control individuals were diagnosed with PTSD during the follow-up period, they were censored from the study as unexposed individuals and were re-entered as exposed individuals who were matched to ten unexposed individuals from the general population on sex, birth year, and county of residence in year of diagnosis.

For sibling analysis, we selected everyone with PTSD from the main study population with at least one full biological sibling who was not diagnosed with PTSD. Therefore, individuals with PTSD in the sibling cohort comprised a subset of the matched cohort. If available, more than one sibling was included to maximise statistical power. Siblings had to be alive at the date of matching, and we adjusted for sex and birth year in the sibling analysis.

Follow-up was done for one dataset linking all registers from which data were used in this study. Each participant was followed up from the date of matching (ie, the index person’s first PTSD diagnosis) until violent crime conviction or until being censored at emigration, death, or Dec 31, 2013, whichever occurred first.

As this was a nationwide, register-based study and individuals were not identifiable at any time, informed consent was waived in accordance with Swedish regulations.^26^

### Outcomes and covariates

The primary outcome was time to violent crime from the date of PTSD diagnosis or the date of the index person’s PTSD diagnosis, referred to as violent crime. Violent crime was defined, in line with previous research,^21^ by convictions of aggravated assault, common assault, homicide (including attempted homicide), arson, robbery, any sexual offense, illegal threats, and intimidation from the National Crime Register. We chose convictions from the National Crime Register as our primary outcome due to its near-complete national coverage,^19^ allowing for an objective measure of violent crime.

Covariates were selected on the basis of previous literature, and we chose covariates that showed particularly strong associations with risk of violence perpetration in individuals with psychiatric disorders,^16,27^ including PTSD, or history of violent crime. Sex data were obtained from the Total Population Register. The Total Population Register collects sex data, recorded as either 1 for male or 2 for female, upon birth or immigration.

To account for socioeconomic factors, and in line with previous research,^27^ we included information on parental income, educational, and immigration background. Data on education and income were obtained from the Integrated Database for Health Insurance and Labour Market Studies, data on death were obtained from the Cause of Death Register, and data on emigration were obtained from the Migration Register. Low family income was defined^27^ by disposable family income in the bottom decile of the population, measured in both biological parents at the end of the year the index person turned 15 years. If these income data were missing, we used data from the end of the year the index person turned 14 years or from the year that data became available. Low parental educational was defined as neither biological parent having secondary-school qualifications, or, if information was missing for one parent, one biological parent having less than secondary-school education. Parental immigration background was defined by at least one biological parent born outside the Nordic countries (ie, Denmark, Finland, Iceland, Sweden, and Norway). For sibling parental characteristics, parental immigration status and parental education were unlikely to vary between siblings, so we only included information on low family income between siblings.

Diagnoses of SUD, schizophrenia, bipolar disorder, major depressive disorder, ADHD, and conduct disorder have been linked to both PTSD^28,29^ and increased risk of violent crime.^30,31^ However, the direction of the effects of psychiatric comorbidities on PTSD and violent crime are unclear. As such, we identified lifetime prevalence of psychiatric comorbidities in people with and without PTSD and violent crime convictions (appendix p 3). We also identified whether these comorbidities preceded PTSD diagnosis. We did not use a diagnostic hierarchy, so individuals could be diagnosed with multiple psychiatric comorbidities. Once diagnosed with a psychiatric comorbidity, this diagnosis was treated as present throughout the lifetime of an individual. For psychiatric comorbidities in individuals without PTSD, we used the date of PTSD diagnosis of the matched diagnosed individual or diagnosed sibling.

We defined history of violent crime as a violent crime conviction before start of follow-up (ie, before the date of first PTSD diagnosis of the index person or the date of matching of the unexposed person). We investigated history of violent crime specifically because previous research has shown violent crime history is a key risk factor for future convictions of violent crime.^16,32^

### Statistical analysis

To investigate the association between PTSD and violent crime in the Swedish general population, we estimated absolute risk using Kaplan-Meier curves. The Kaplan-Meier estimate of the proportion with the outcome can be interpreted as a cumulative incidence, which ignores the competing outcome event of death. We used stratified Cox regression (the conditional, on cluster, version of Cox regression) to evaluate the associations between PTSD and violent crime. Therefore, any covariates shared within the strata are adjusted by design, regardless of whether they are measured. In the matched cohort, this corresponds to adjusting for matching variables (ie, birth year, sex, and region) and in the sibling cohort, this corresponds to all familial factors shared by siblings (ie, parental factors, socioeconomic status, home environment, and genetics [on average, 50% of segregating alleles]).

Associations were expressed as hazard ratios (HRs) with 95% CIs. In the stratified Cox regression, comparisons were made within each matched set (ie, each individual diagnosed with PTSD and their ten matched control individuals), allowing for varying baseline hazard rates across strata. We first fitted a minimally adjusted model (model 1), adjusting for sex, birth year, and county of residence in the year of the index person’s first PTSD diagnosis through matching. We subsequently adjusted for parental characteristic data (model 2) and then for psychiatric comorbidities diagnosed before PTSD (model 3). Finally, we estimated HRs in the fully adjusted model stratified by SUD (model 4) and by history of violent crime (model 5).

To estimate the extent to which an association observed at the population level could be accounted for by shared familial factors, we conducted sibling control analysis, comparing the risk of committing a violent crime in siblings differentially exposed to PTSD. We conducted a stratified Cox regression, including each family as separate strata. We initially adjusted for sex and birth year of each sibling, before also adjusting for low family income and previous psychiatric comorbidities. We did not adjust for parental immigration status or education, as neither is likely to vary between siblings. We also estimated HRs stratified by SUD and by history of violent crime.

We used Kaplan-Meier curves to investigate the absolute risk for violent convictions in individuals with different histories of violence and SUD. We used lifetime prevalence of SUD (ie, both before and after PTSD diagnosis), as it would be the most informative data of risk of recurrence in forensic settings.

We conducted sex-stratified analyses to evaluate potential sex differences. To evaluate potential cohort effects and poor PTSD-diagnosis coverage in the oldest birth cohorts, we restricted the sample to individuals born since 1973 (aged at least 15 years when ICD-9 was introduced in Sweden) and then since 1983 (aged at least 15 years when ICD-10 was introduced in Sweden). We then conducted analyses excluding individuals previously diagnosed with psychiatric comorbidities to evaluate potential differences in the association between PTSD and violent crime. To evaluate whether the association between PTSD and violent crime differs between convicted and suspected crime, we used data from the National Criminal Suspects Register to conduct sensitivity analyses with violent crime suspicion as the outcome.^33^ This register includes individuals suspected of violent crime after investigation by police, customs authorities, or prosecution services from 1995 to 2016.^34,35^ We defined violent crime suspicion using the same definition as convictions (ie, suspicion of aggravated assault, common assault, homicide [including attempted homicide], arson, robbery, any sexual offense [including child pornography offenses], illegal threats, and inti-midation). Finally, as the cause of PTSD diagnoses are not reported in the National Patient Register, we conducted an age-at-diagnosis sensitivity analysis in the matched cohort for people diagnosed with PTSD before age 18 years and those diagnosed aged 18 years or older as a proxy for childhood-related PTSD and combat-related or work-related PTSD.

As the Total Population Register, the National Patient Register, the National Crime Register, the Multi-Generation Register, the Cause of Death Register, and the Migration Register have near-complete national coverage, there is minimal missing data and loss to follow-up. As standard in Cox regression, all individuals contributed follow-up time until censoring at the date of the event, death, emigration, or end of follow-up, whichever came first.

Linkage of the registers and overall preparation of the dataset used for statistical analyses were done with SAS version 9.4 and statistical analyses were conducted via the survival package in R version 4.0.5.^36,37^

### Role of the funding source

There was no funding source for this study.

## Results

3 890 765 individuals born in Sweden between 1958 and 1993 were assessed for eligibility for inclusion in this study. After individuals who died or emigrated before their 15th birthday, were adopted, were twins, or whose biological parents could not be identified were excluded, 3 597 445 eligible participants were identified, of whom 13 119 individuals were diagnosed with PTSD (appendix p 13). 131 190 matched control individuals from the general population who were alive, living in Sweden, and without a PTSD diagnosis at the date of matching were also included, resulting in a total matched cohort population of 144 309. In the sibling cohort, the final sample included in the analysis from 3 597 445 eligible participants was 9114 individuals with PTSD and 14 613 full biological siblings without PTSD. 9113 of these individuals with PTSD and 833 of these siblings without PTSD were also included in the matched cohort. 338 (0·3%) of 131 190 unexposed matched control individuals were diagnosed with PTSD during the follow-up period.

In the matched cohort, 9856 (75·1%) of 13 119 participants with PTSD were female and 3263 (24·9%) were male (table 1). Mean follow-up was 5*·*6 years (SD 5·1, range 0–27). In the sibling cohort, 6956 (76·3%) of 9114 participants with PTSD were female and 2158 (23·7%) were male. Mean follow-up was 5*·*6 years (SD 5*·*1, range 0*·*0–27*·*0). 588 (0·4%) of 144 309 participants in the matched cohort and 69 (0·3%) of 23 727 participants in the sibling cohort were excluded due to missing parental characteristic data. Descriptive data of included and excluded individuals suggested minimal differences between groups (appendix p 4).

**Figure 1:**
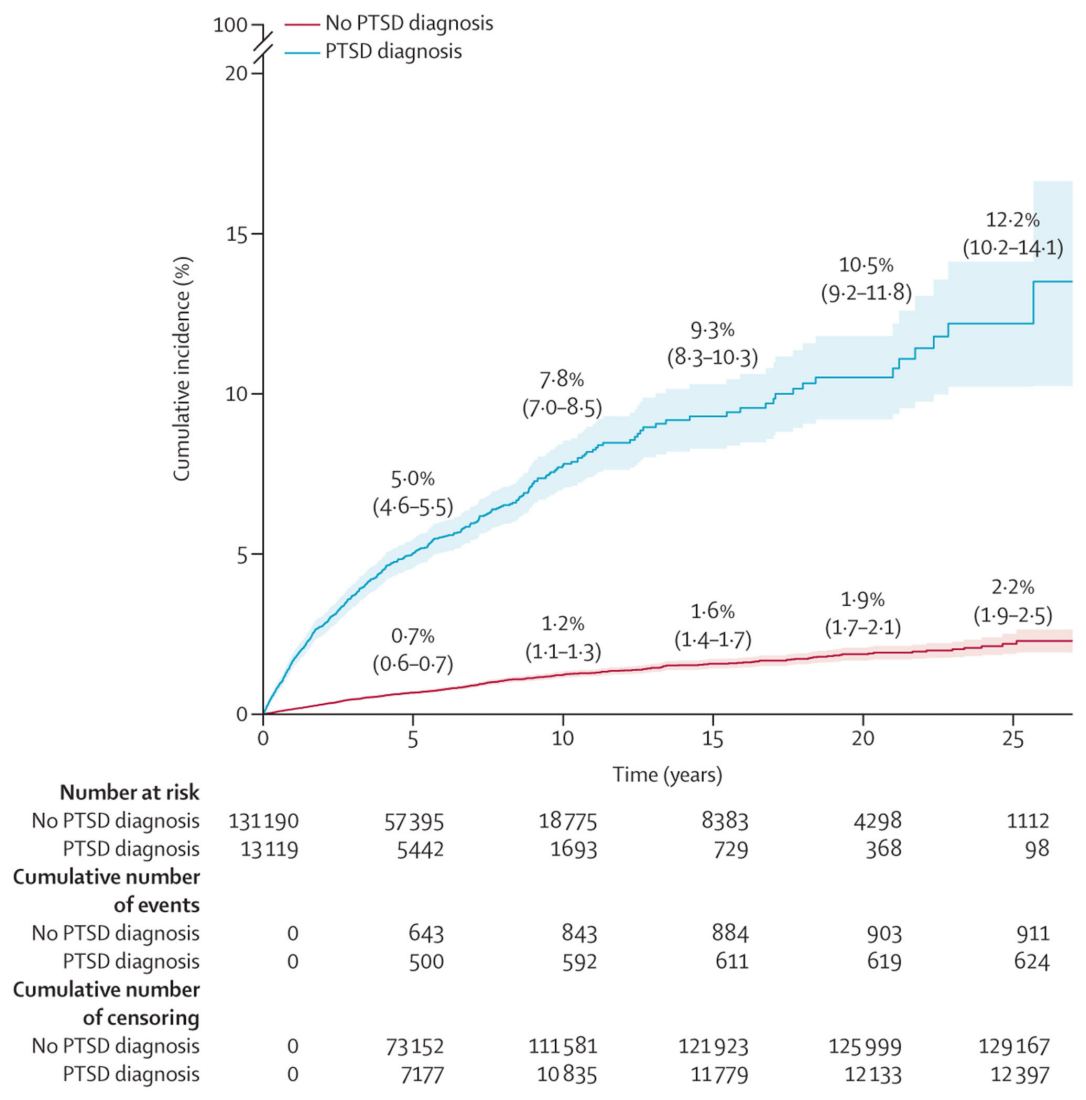
Cumulative incidence of violent crime conviction over time from start of follow-up in the matched cohort (n=144 309) PTSD=post-traumatic stress disorder.

The cumulative incidence of violent crime conviction after 5 years was 5*·*0% (95% CI 4·6–5·5) in individuals diagnosed with PTSD versus 0·7% (0·6–0·7) in individuals without PTSD. At the end of follow-up, the cumulative incidence was 13*·*5% (11·3–16·6) in individuals diagnosed with PTSD versus 2*·*3% (1·9–2·6) in individuals without PTSD (figure 1). For cumulative incidence of violent crime conviction per year, see the appendix (p 5). In the minimally adjusted model (model 1), individuals with PTSD were significantly more likely to be convicted of a violent crime than individuals without PTSD (table 2). Even after adjusting for parental characteristics and previous psychiatric comorbidities (model 3), the risk remained significantly increased (table 2).

In our analysis stratified by SUD (model 4), individuals with no previous SUD and PTSD had a significantly greater risk of violent crime conviction relative to individuals without PTSD and without SUD; individuals with previous SUD and PTSD also had a significantly greater risk of violent crime conviction than those without PTSD but with a previous SUD, although the HR was lower than for those with no previous SUD. Individuals with no history of violent crime and PTSD had a significantly higher risk of violent crime conviction relative to individuals without PTSD and no history of violent crime; individuals with a history of violent crime and PTSD also had a higher risk of violent crime conviction than those without PTSD, although the HR was lower than for those with no history of violent crime.

We investigated the proportionality of hazards, and although there were some deviations (appendix p 14), we deemed the assumption of proportionality suitable for analyses.

In the sibling cohort analysis, after adjusting for sex, birth year, and family income, siblings diagnosed with PTSD had a significantly higher risk of violent crime conviction than their siblings without PTSD. The risk remained increased even in siblings with PTSD without SUD and without history of violent crime (table 2).

**Figure 2:**
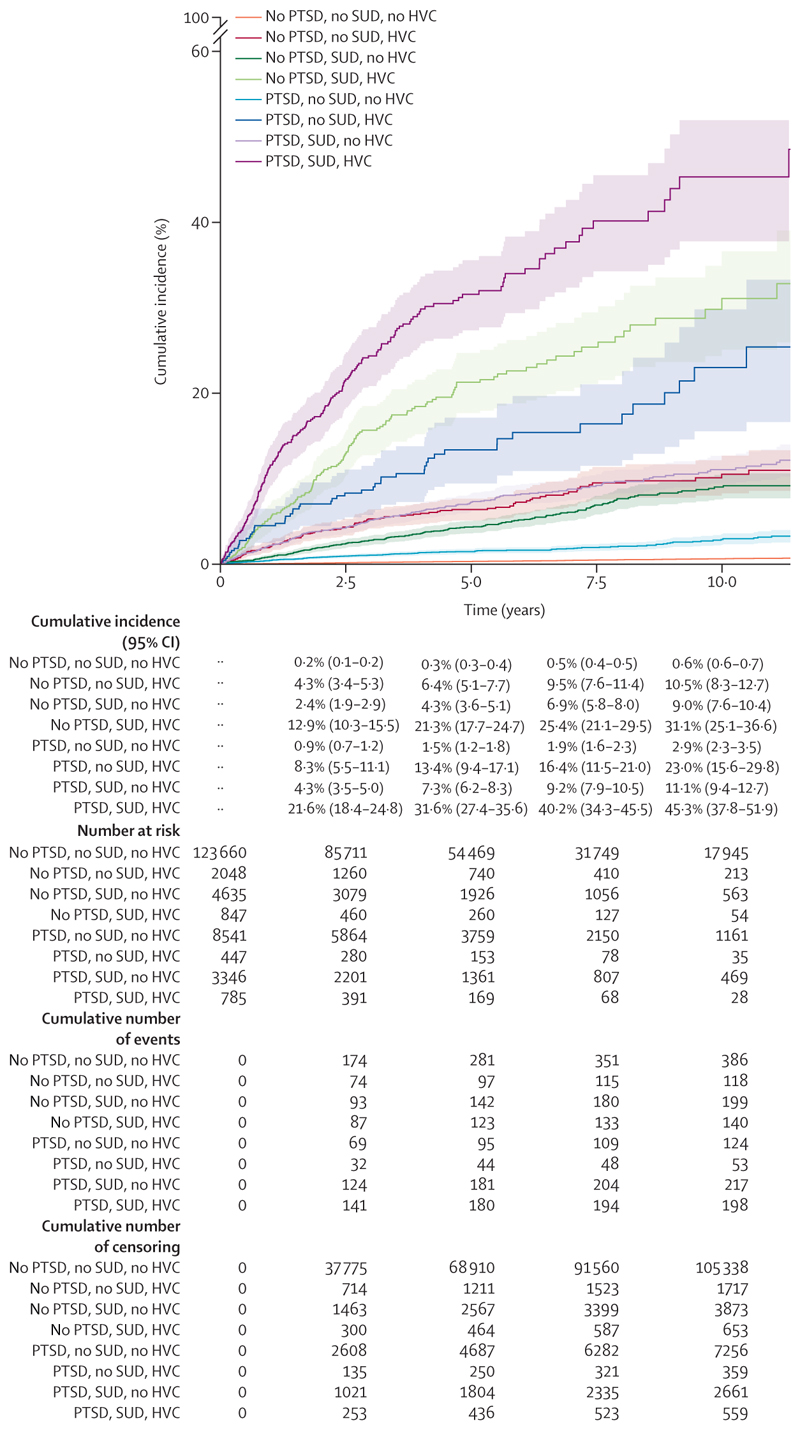
Cumulative incidence of violent crime conviction in the matched cohort (n=144 309) by PTSD, lifetime SUD prevalence, and HVC conviction HVC=history of violent crime. PTSD=post-traumatic stress disorder. SUD=substance use disorder.

Individuals with a PTSD diagnosis but no history of violent crime or SUD, our largest subgroup, had a cumulative incidence of violent crime conviction of 2·9% (95% CI 2·3–3·5) at 10-year follow-up (figure 2). Cumulative incidence of violent crime in individuals with a history of violent crime and SUD at 10-year follow-up ranged from 31·1% (25·1–36·6) in those without PTSD to 45·3% (37·8–51·9) in those with PTSD (figure 2). For cumulative incidences of violent crime conviction stratified by PTSD diagnosis, lifetime SUD, and history of violent crime conviction per year, see the appendix (p 6).

The HR of violent crime conviction for individuals with PTSD was similar for male and female participants (appendix p 7). In terms of violent crime suspicion, 1611 (12·3%) of 13 119 individuals with PTSD and 3005 (2*·*3%) of 131 190 matched control individuals without PTSD were suspected of a violent crime (appendix p 8). Making suspicion of violent crime the outcome, restricting the birth cohorts, and excluding individuals with previous psychiatric comorbidities all provided similar results to the original matched cohort, with only slight attenuation in relative risk (appendix pp 9–11). Separate analyses by age at diagnosis also provided similar results as the main analyses, with overlapping 95% CIs for all estimates between age groups (appendix p 12).

## Discussion

In this nationwide, register-based cohort study, we investigated the association between PTSD and time to violent crime by analysing national population data with information on siblings and up to 27 years of follow-up, and found that PTSD diagnosis is associated with increased risk of violent crime conviction. The risk remained increased even after adjusting for parental characteristics, psychiatric comorbidities (including previous SUD), history of violent crime, and familial confounders (both genetic and environmental) shared by siblings. However, this interpretation should be made with caution as this study is observational, and we cannot definitively exclude residual confounding (eg, by environmental and genetic factors not shared by siblings).

Overall, our results are consistent with previous studies investigating PTSD and violent crime in military veterans^11^ and previous research showing an association between PTSD and violent offending in current non-military samples.^13^ Our sample was ten times larger than the previous largest study,^38^ and we showed a significantly higher risk of violent crime conviction in individuals with PTSD than in individuals without PTSD. The risk was attenuated but remained increased after controlling for measured factors linked to both PTSD and increased risk of violent crime and unmeasured genetic and environmental familial factors. Therefore, the hypothesis that PTSD might increase the risk of violent crime is strengthened by our results. However, less than 5% of individuals with PTSD were convicted of a violent crime; the absolute risk of violence in this vulnerable population remains low and our analyses highlight other factors, such as SUD and history of violent crime, contributing to increased risk of violent acts and convictions.

We note that individuals with PTSD had increased rates of all considered psychiatric comorbidities, including early onset disorders on the externalising spectrum (eg, ADHD and conduct disorder), as well as mood disorders and psychotic disorders. However, adjusting for these disorders only had minor effects on the association between PTSD and violent crime, suggesting that SUD and history of violent crime are more salient modifiers of the association.

PTSD is characterised by dysregulation in various stress-mediating biological systems in response to trauma,^6^ including in the neuroendocrine hypothalamus–pituitary–adrenal (HPA) axis,^39^ which regulates physiological stress responses^40^ and is responsible for the fight-or-flight reaction to stress. Regarding the behavioural implications of PTSD-related HPA axis dysregulation, this study only investigated the fight reaction, and our data support the hypothesis of this mechanism relating PTSD and risk of violent crime.

Because of the non-trivial, general-population prevalence of PTSD and the strong increased risk of violent crime convictions observed in this study, our results have direct implications for public health. Both targeted health-care initiatives supporting people diagnosed with PTSD on an individual level^41^ and large-scale PTSD prevention efforts,^42^ especially for vulnerable groups (eg, individuals exposed to natural disasters or large-scale accidents), could aim to reduce violent-crime risk.^9^

History of violent crime and SUD, as shown in previous research,^16^ are key risk factors for violent crime. In our analyses, these factors showed a high absolute risk and hazard ratios, even in the absence of PTSD, but risk of violent crime conviction was further increased among people with a PTSD diagnosis compared with individuals without PTSD and compared with siblings without PTSD. This increase suggests that individuals with a history of violent crime and SUD should be supported through intervention, and that a diagnosis of PTSD in this already vulnerable group might warrant extra prudence. However, the interpretation of findings in these subgroups should be cautious as the numbers of individuals in our analysis stratified by history of violent crime with SUD or PTSD were relatively small.

This study has several strengths, including a large and population-representative dataset, longitudinal follow-up, and objective measures of PTSD and violent crime through register data.

There are also several limitations. First, Swedish register data do not include information about the traumatic event or events leading to the PTSD diagnosis. Therefore, whether the type of trauma preceding PTSD could moderate the association between PTSD and violent crime is unclear. As such, we could not establish whether adverse childhood experiences, which have been linked to children involved with the criminal justice system in the USA^43^ and increased risk of violent behaviour in adulthood,^44^ might have contributed. However, we found similar risk of violent crime in individuals diagnosed with PTSD before and after age 18 years. Further research should investigate the association between PTSD and violent crime in populations in whom adverse childhood experiences (especially adverse childhood experiences involving criminal justice systems) can be identified.

Second, we focused on PTSD as diagnosed and treated in specialist care as the National Patient Register does not include information about the primary care settings and provides less coverage of private health care,^24^ and violent crime as indicated by convictions. Although the registers provide objective measures, this will have also resulted in primarily including the more extreme instances of PTSD and, to some extent, violence. Swedish data suggest that while only a small proportion of offences are reported overall, threats are the most reported and robbery is the least reported.^45^ Whether our results can be generalised to less severe or undetected PTSD and other, less extreme or unreported forms of antisocial behaviour is uncertain. Less severe PTSD and unmeasured antisocial behaviour remain a target for future research (eg, assessed via survey data).

Third, PTSD diagnoses might be under-represented in earlier birth cohorts due to outpatient register data only being available from 2001 onwards.^24^ To address this, we conducted sensitivity analyses in two younger birth cohorts (individuals born in 1973 and 1983) with improved coverage of PTSD diagnosis, which showed similar results to original analyses.

Fourth, there could be differences in the likelihood of being convicted once a crime has been committed between individuals with PTSD compared with individuals without PTSD.^46^ Similarly, violent-crime conviction could increase the likelihood of being diagnosed with PTSD as one enters the correctional system. However, our sensitivity analyses using data on suspicions of violent crime, which would be less affected by such potential reverse causation, provided similar results to our original analysis using convictions. This similarity in results suggests that reverse causality is unlikely to be the primary explanation of our findings.

Finally, the date of PTSD diagnosis in the National Patient Register does not necessarily correspond to the age of onset for psychiatric symptoms; individuals might wait more than a decade before seeking treatment.^47^ Therefore, we might have misclassified some individuals with active but undiagnosed PTSD as unexposed. However, this misclassification would only lead to attenuated associations.

Our study showed a strong association between PTSD and violent crime both in the Swedish general population and in comparison with sibling control individuals. PTSD might be considered a potential risk factor for violent crime and should be a target of prevention, with the potential to reduce crime rates in this vulnerable population.

## Supplementary Material

Supplementary Tables and Figures

## Figures and Tables

**Table 1:** Baseline characteristics for the matched and sibling cohorts

	Matched cohort (n=144 309)			Sibling cohort (n=23 727)	
	PTSD	No PTSD		PTSD	No PTSD
Participants	13 119/144 309 (9·1%)	131 190/144 309 (90·9%)		9114/23 727 (38·4%)	14 613/23 727 (61·6%)
Median age at diagnosis, years	29·9 (22·5–39·1)	··		30·0 (22·8–38·9)	··
Mean follow-up time, years	5·3 (5·0)	5·6 (5·2)		5·4 (5·0)	5·6 (5·1)
Median follow-up time, years	4·0 (1·8–7·3)	43 (2·0–7·6)		4·2 (1·8–7·4)	4·3 (2·0–7·6)
Total follow-up time, person-years	70 044	738 296		49 411	82 402
Sex					
Male	3263/13 119 (24·9%)	32 630/131 190 (24·9%)		2158/9114 (23·7%)	7773/14 613 (53·2%)
Female	9856/13 119 (75·1%)	98 560/131 190 (75·1%)		6956/9114 (76·3%)	6840/14 613 (46·8%)
Parental characteristics					
Parental immigration	1379/13 119 (10·6%)	9828/131 190 (7·5%)		··	··
Low parental education	2106/13 119 (16·1%)	18 517/131 190 (14·1%)		··	··
Low family income	1091/13 119 (8·3%)	9442/131 190 (7·2%)		701/9114 (7·7%)	1298/14 613 (8·9%)
Missing parental information*	81/13 119 (0·6%)	507/131 190 (0·4%)		28/9114 (0·3%)	41/14 613 (0·3%)
Psychiatric comorbidities before PTSD diagnosis					
Substance use disorder	2711/13 119 (20·7%)	3662/131 190 (2·8%)		1861/9114 (20·4%)	899/14 613 (6·2%)
Schizophrenia	119/13 119 (0·9%)	322/131 190 (0·2%)		84/9114 (0·9%)	85/14 613 (0·6%)
Bipolar disorder	738/13 119 (5·6%)	765/131 190 (0·6%)		583/9114 (6·4%)	152/14 613 (1·0%)
Major depressive disorder	5205/13 119 (39·7%)	4847/131 190 (3·7%)		4045/9114 (44·4%)	1060/14 613 (7·3%)
ADHD and conduct disorder	1013/13 119 (7·7%)	563/131 190 (0·4%)		686/9114 (7·5%)	153/14 613 (1·0%)
History of violent crime	1232/13 119 (9·4%)	2895/131 190 (2·2%)		758/9114 (8·3%)	1008/14 613 (6·9%)
Violent crime during follow-up					
Convicted of violent crime	625/13 119 (4·8%)	912/131 190 (0·7%)		388/9114 (4·3%)	360/14 613 (2·5%)
Age at crime, years	29·6 (23·1–38·4)	28·4 (22·0–38·6)		30·0 (24·0–38·2)	27·2 (21·5–37·2)

Data are n/N (%), mean (SD), or median (IQR). ADHD=attention-deficit hyperactivity disorder. PTSD=post-traumatic stress disorder. *588 participants in the matched cohort and 69 participants in the sibling cohort were excluded due to missing information on parental characteristics.

**Table 2:** Hazard ratios for stratified Cox regression

	Matched cohort				Sibling cohort		
	PTSD	Total	HR (95% CI)		PTSD	Total	HR (95% CI)
Model 1	13 119	144 309	7·4 (6·6–8·2)		9114	23 727	3·2 (2·6–3·9)
Model 2	13 038	143 721	7·4 (6·6–8·2)		··	··	··
Model 3	13 038	143 721	6·4 (5·7–7·2)		9086	23 658	3·2 (2·6–4·0)
Model 4							
No previous SUD diagnosis	10 350	137 396	6·1 (5·3–6·9)		7233	20 907	3·1 (2·4–4·1)
Previous SUD diagnosis	2688	6325	1·9 (1·5–2·6)		1853	2751	1·7 (1·1–2·7)
Model 5							
No history of violent crime	11 826	139 641	5·0 (4·3–5·7)		8333	21 901	3·0 (2·3–3·9)
History of violent crime	1212	4080	2·2 (1·7–3·0)		753	1757	2·0 (1·2–3·2)

Data are n/N (%) or HR (95% CI). Analyses include each matched set as separate strata in the matched cohort and each family as separate strata in the sibling cohort. All models in the matched cohort are implicitly adjusted for sex, birth year, and county of residence in year of diagnosis. All models in the sibling cohort are adjusted for sex and birth year. The minimally adjusted model (model 1) adjusts for sex, birth year, and county of residence in year of diagnosis through matching. Model 2 also adjusts for parental characteristics. The fully adjusted model (model 3) also adjusts for parental characteristics and psychiatric comorbidities (ie, major depressive disorder, schizophrenia, bipolar disorder, ADHD, and conduct disorder) diagnosed before PTSD diagnosis. For siblings, parental characteristics only include parental low income. For psychiatric comorbidities in individuals without PTSD, we used the date of PTSD diagnosis of the matched diagnosed individual or diagnosed sibling. Model 4 is fully adjusted (adjusting for parental characteristics and psychiatric comorbidities) and stratified by previous SUD diagnosis. Model 5 is fully adjusted (adjusting for parental characteristics and psychiatric comorbidities) and stratified by history of violent crime, and includes adjustment for previous SUD. ADHD=attention-deficit hyperactivity disorder. HR=hazard ratio. PTSD=post-traumatic stress disorder. SUD=substance use disorder.

## Data sharing

The Public Access to Information and Secrecy Act in Sweden prohibits individual-level data being publicly available. Researchers who are interested in replicating this study can apply for individual-level data through Statistics Sweden (https://www.scb.se/en/services/ordering-data-and-statistics/ordering-microdata/). Data on criminal convictions for research purposes can be requested from the National Crime Register (https://bra.se/bra-in-english/home/about-bra/contact-us.html). Data on patient health can be applied for through Socialstyrelsen (https://www.socialstyrelsen.se/en/statistics-and-data/registers/). Analysis coding and aspects of the protocol can be available by request to the corresponding author.
