# Supplementary material for: Post-traumatic stress disorder and the risk of violent crime conviction in Sweden: a nationwide, register-based cohort study
Source: Lancet Public Health. Author manuscript; Available in PMC 2025 Sep 2. (PMC7618061; doi:10.1016/S2468-2667(23)00075-0)
Supplement: Supplementary Tables and Figures [file EMS208248-supplement-Supplementary_Tables_and_Figures.pdf]

Supplementary Online Content

Table S1. ICD-8, ICD-9, and ICD-10 codes for defining comorbid psychiatric disorders from the Swedish National Patient Register (PAGE 2)

Table S2. Lifetime prevalence of psychiatric comorbidities (PAGE 3)

Table S3. Descriptives for excluded versus non-excluded individuals (PAGE 4)

Table S4. Percentage cumulative incidence of violent crime (95% confidence interval) (PAGE 5).

Table S5. Percentage cumulative incidence of violent crime stratified on PTSD, SUD, and violent crime conviction history (95% confidence interval) (PAGE 6).

Table S6. Hazard ratios for sex-stratified models in the matched cohort (PAGE 7)

Table S7. Descriptives for individuals suspected of violent crime in the matched cohort, as defined by the National Criminal Suspects Register (PAGE 8)

Table S8. Hazard ratios for stratified Cox regression using suspicion of violent crime as outcome (PAGE 9)

Table S9. Hazard ratios for sensitivity analysis in the restricted birth matched cohort, including data for stratifying by SUD and history of violent crime (PAGE 10)

Table S10. Hazard ratios for stratified Cox regression excluding individuals with psychiatric comorbidities preceding PTSD diagnosis (PAGE 11)

Table S11. Hazard ratios for stratified Cox regression, separately for those first diagnosed with PTSD prior to age 18 and those diagnosed age 18 or older in the matched cohort (PAGE 12)

Figure S1. Matched and sibling cohorts, as obtained from the Swedish population (PAGE 13)

Figure S2. Assessment of non-proportional hazards of violent crime over follow-up time (PAGE 14)

Table S1. ICD-8, ICD-9, and ICD-10 codes for defining comorbid psychiatric disorders from the Swedish National Patient Register

	**ICD-8 (1981-1986)**	**ICD-9 (1987-1996)**	**ICD-10 (1997-)**
Substance use disorder	303, 304	303, 304, 305A, 305X	F10-F19, excluding F17
Depression	296·2, 300·4	296B, 300E, 311	F32-F39, excluding 32·3
Bipolar disorder	296, excluding 296·2	296, excluding 296B	F30-F31
Schizophrenia	295	295	F20
ADHD and CD	N/A	312, 314	F90-F91

Abbreviations: ICD = International Classification of Diseases, ADHD = attention deficit hyperactivity disorder, CD = conduct disorder.

Table S2. Lifetime prevalence of psychiatric comorbidities

	**Matched Cohort**		**Sibling Cohort**	
	**PTSD** ***(% total)***	**No PTSD** ***(% total)***	**PTSD** ***(% total)***	**No PTSD** ***(% total)***
**TOTAL**	13 119 *(9·1%)*	131 190 *(90·9%)*	9 114 *(38·5%)*	14 613 *(61·5%)*
	**PTSD** ***(% with PTSD)***	**No PTSD** ***(% without PTSD)***	**PTSD** ***(% with PTSD)***	**No PTSD** ***(% without PTSD)***
Substance use disorder	4 131 *(31·5%)*	5 482 *(4·2%)*	2 778 *(30·5%)*	1 312 *(9·0%)*
Schizophrenia	258 *(2·0%)*	411 *(0·3%)*	172 *(1·9%)*	120 *(0·8%)*
Bipolar disorder	1 692 *(12·9%)*	1 467 *(1·1%)*	1 201 *(13·2%)*	295 *(2·0%)*
Major depressive disorder	8 160 *(62·2%)*	8 546 *(6·5%)*	5 694 *(62·5%)*	1 725 *(11·8%)*
ADHD and CD	2 101 *(16·0%)*	2 116 *(1·6%)*	1 348 *(14·8%)*	552 *(3·8%)*
	**Violent crime conviction** ***(% convicted)***	**No violent crime conviction** ***(% not convicted)***	**Violent crime conviction** ***(% convicted)***	**No violent crime conviction** ***(% not convicted)***
Substance use disorder	795 *(51·7%)*	8 818 *(6·2%)*	445 *(59·5%)*	3 645 *(15·9%)*
Schizophrenia	53 *(3·4%)*	616 *(0·4%)*	33 *(4·4%)*	259 *(1·1%)*
Bipolar disorder	115 *(7·5%)*	3 044 *(2·1%)*	66 *(8·8%)*	1 430 *(6·2%)*
Major depressive disorder	556 *(36·2%)*	16 150 *(11·3%)*	315 *(42·1%)*	7 104 *(30·9%)*
ADHD and CD	321 *(20·9%)*	3 896 *(2·7%)*	181 *(24·2%)*	1 719 *(7·5%)*

Abbreviations: PTSD = post-traumatic stress disorder, ADHD = attention deficit hyperactivity disorder, CD = conduct disorder. Entries reflect column percent (number of individuals with or without PTSD diagnosis, then amount of individuals who were or were not convicted of a violent crime during follow-up). Psychiatric comorbidities were not diagnostically hierarchical, meaning that individuals could be diagnosed with multiple psychiatric comorbidities. Once an individual was diagnosed with a psychiatric comorbidity, this diagnosis was treated as lifetime.

Table S3. Descriptives for excluded versus non-excluded individuals

	Not excluded		Excluded	
	**PTSD** *(% total)*	**No PTSD** *(% total)*	**PTSD** *(% total)*	**No PTSD** *(% total)*
**TOTAL**	13 038 *(9·1%)*	130 683 *(90·9%)*	81 *(13·8%)*	507 *(86·2%)*
Mean age at diagnosis (Median) [interquartile range]	31·0 (29·9) (22·5 – 39·0)	..	40·2 (43·9) (32·9 – 48·7)	..
Mean follow-up time in years (standard deviation) [Min–Max]	5·3 (5·0) [0·0 – 27·0]	5·6 (5·1) [0·0 – 27·0]	6·4 (6·5) [0·0 – 26·3]	9·0 (6·0) [0·0 – 26·7]
Median follow-up time in years (interquartile range)	4·0 (1·8 – 7·2)	4·2 (2·0 – 7·6)	3·9 (2·3 – 8·1)	5·6 (2·6 – 9·7)
Follow-up time, person-years	69 524	734 727	519	3 570
**Sex**				
Male	3 225 *(24·7%)*	32 483 *(24·9%)*	38 *(46·9%)*	147 *(29·0%)*
Female	9 813 *(75·3%)*	98 200 *(75·1%)*	43 *(53·1%)*	360 *(71·0%)*
**Parental characteristics**				
Parental immigration	1 370 *(10·5%)*	9 776 *(7·5%)*	9 (11·1%)	52 (10·3%)
Low parental education	2 075 *(15·9%)*	18 293 *(14·0%)*	31 (38·3%)	224 (44·2%)
Low family income	1 083 *(8·3%)*	9 412 *(7·2%)*	8 *(9·9%)*	30 *(5·9%)*
Missing parental information	0 *(0·0%)*	0 *(0·0%)*	81 *(100·0%)*	507 *(100·0%)*
**Psychiatric comorbidities (prior to PTSD diagnosis)**				
Substance use disorder	2 688 *(20·6%)*	3 637 *(2·8%)*	23 *(28·4%)*	25 *(4·9%)*
Schizophrenia	115 *(0·9%)*	319 *(0·2%)*	5 or less*	5 or less*
Bipolar disorder	733 *(5·6%)*	733 *(0·6%)*	5 or less*	5 or less*
Major depressive disorder	5 168 *(39·6%)*	4 816 *(3·7%)*	37 *(45·7%)*	31 *(6·1%)*
ADHD and CD	1 009 *(7·7%)*	561 *(0·4%)*	5 or less*	5 or less*
**History of violent crime**				
History of violent crime	1 212 *(9·3%)*	2 868 *(2·2%)*	20 *(24·7%)*	27 *(5·3%)*
**Violent crime during follow-up**				
Convicted of violent crime	620 *(4·8%)*	901 *(0·7%)*	5 or less*	11 *(2·2%)*
Median age at crime in years (interquartile range)	29·6 (23·1 – 38·4)	28·3 (22·0 – 38·4)	30·1 (21·1 – 49·0)	40·5 (23·1 – 42·0)

Abbreviations: PTSD = post-traumatic stress disorder, ADHD = attention deficit hyperactivity disorder, CD = conduct disorder. Psychiatric comorbidities were not diagnostically hierarchical, meaning that individuals could be diagnosed with multiple psychiatric comorbidities. Once an individual was diagnosed with a psychiatric comorbidity, this diagnosis was treated as lifetime. For psychiatric comorbidities in individuals without PTSD, we used the matched diagnosed individual's (or diagnosed sibling's) date of PTSD diagnosis. For sibling parental characteristics, parental immigration status and parental education is unlikely to vary between siblings, so we only included information on low family income between siblings. Excluded due to missing information on parental characteristics: 588 (matched cohort), 69 (sibling cohort).

*Due to Swedish data privacy law, we are not able to show numerical data for categories of 5 or less individuals.

Table S4. Percentage cumulative incidence of violent crime (95% confidence interval).

**Time since diagnosis**	**1 years**	**2 years**	**3 years**	**4 years**	**5 years**
No PTSD diagnosis	0·2% (0·1-0·2)	0·3% (0·3-0·3)	0·5% (0·4-0·5)	0·6% (0·5-0·6)	0·7% (0·6-0·7)
PTSD diagnosis	1·7% (1·5-1·9)	2·8% (2·5-3·1)	3·7% (3·4-4·1)	4·5% (4·1-4·9)	5·0% (4·6-5·5)
	**6 years**	**7 years**	**8 years**	**9 years**	**10 years**
No PTSD diagnosis	0·8% (0·7-0·8)	0·9% (0·8-1·0)	1·0% (1·0-1·1)	1·1% (1·0-1·2)	1·2% (1·1-1·3)
PTSD diagnosis	5·5% (5·1-6·0)	6·0% (5·4-6·5)	6·5% (5·9-7·1)	7·2% (6·5-7·8)	7·8% (7·0-8·5)
	**11 years**	**12 years**	**13 years**	**14 years**	**15 years**
No PTSD diagnosis	1·3% (1·2-1·4)	1·4% (1·3-1·5)	1·4% (1·3-1·6)	1·5% (1·4-1·7)	1·6% (1·4-1·7)
PTSD diagnosis	8·3% (7·5-9·0)	8·5% (7·6-9·3)	9·0% (8·0-9·9)	9·2% (8·2-10·2)	9·3% (8·3-10·3)
	**16 years**	**17 years**	**18 years**	**19 years**	**20 years**
No PTSD diagnosis	1·6% (1·5-1·8)	1·7% (1·5-1·8)	1·7% (1·6-1·9)	1·8% (1·6-2·0)	1·9% (1·7-2·1)
PTSD diagnosis	9·6% (8·5-10·6)	9·7% (8·6-10·8)	10·3% (9·1-11·6)	10·5% (9·2-11·8)	10·5% (9·2-11·8)
	**21 years**	**22 years**	**23 years**	**24 years**	**25 years**
No PTSD diagnosis	1·9% (1·7-2·1)	2·0% (1·7-2·2)	2·0% (1·8-2·3)	2·1% (1·8-2·4)	2·2% (1·9-2·5)
PTSD diagnosis	10·8% (9·4-12·2)	11·4% (9·8-13·1)	12·2% (10·2-14·1)	12·2% (10·2-14·1)	12·2% (10·2-14·1)

Abbreviations: PTSD = post-traumatic stress syndrome, SUD = substance use disorder.

Table S5. Percentage cumulative incidence of violent crime stratified on PTSD, SUD, and violent crime conviction history (95% confidence interval).

**Time since diagnosis**	**1 years**	**2 years**	**3 years**	**4 years**	**5 years**
No PTSD, No SUD, No crime history	0·1% (0·1-0·1)	0·1% (0·1-0·1)	0·2% (0·2-0·2)	0·3% (0·2-0·3)	0·3% (0·3-0·4)
No PTSD, No SUD, Crime history	2·1% (1·4-2·7)	3·8% (2·9-4·7)	5·3% (4·2-6·4)	6·0% (4·7-7·1)	6·4% (5·1-7·7)
No PTSD, SUD, No crime history	0·9% (0·6-1·1)	1·9% (1·5-2·4)	2·8% (2·3-3·3)	3·6% (3·0-4·2)	4·3% (3·6-5·1)
No PTSD, SUD, Crime history,	5·4% (3·8-7·0)	10·6% (8·2-12·8)	15·7% (12·8-18·5)	18·5% (15·2-21·6)	21·3% (17·7-24·7)
PTSD, No SUD, No crime history	0·4% (0·3-0·6)	0·8% (0·6-1·0)	1·1% (0·8-1·3)	1·4% (1·1-1·6)	1·5% (1·2-1·8)
PTSD, No SUD, Crime history	4·5% (2·5-6·5)	7·1% (4·5-9·6)	8·7% (5·8-11·5)	10·6% (7·3-13·8)	13·4% (9·4-17·1)
PTSD, SUD, No crime history	2·2% (1·7-2·7)	3·9% (3·2-4·6)	5·2% (4·3-6·0)	6·2% (5·3-7·1)	7·3% (6·2-8·3)
PTSD, SUD, Crime history	11·6% (9·2-13·9)	17·4% (14·5-20·3)	24·4% (20·9-27·7)	29·9% (25·8-33·7)	31·6% (27·4-35·6)
	**6 years**	**7 years**	**8 years**	**9 years**	**10 years**
No PTSD, No SUD, No crime history	0·4% (0·3-0·4)	0·4% (0·4-0·5)	0·5% (0·5-0·6)	0·6% (0·5-0·6)	0·6% (0·6-0·7)
No PTSD, No SUD, Crime history	7·3% (5·8-8·7)	8·5% (6·8-10·1)	9·5% (7·6-11·4)	9·8% (7·8-11·7)	10·5% (8·3-12·7)
No PTSD, SUD, No crime history	5·2% (4·3-6·0)	6·1% (5·1-7·0)	7·7% (6·5-8·8)	8·3% (7·0-9·6)	9·0% (7·6-10·4)
No PTSD, SUD, Crime history,	22·6% (18·8-26·2)	24·4% (20·3-28·2)	26·6% (22·0-30·9)	28·8% (23·7-33·5)	31·1% (25·1-36·6)
PTSD, No SUD, No crime history	1·6% (1·3-2·0)	1·8% (1·5-2·2)	2·1% (1·7-2·5)	2·5% (2·0-3·0)	2·9% (2·3-3·5)
PTSD, No SUD, Crime history	15·4% (10·9-19·7)	15·4% (10·9-19·7)	16·4% (11·5-21·0)	20·1% (13·7-25·9)	23·0% (15·6-29·8)
PTSD, SUD, No crime history	8·2% (7·0-9·4)	8·8% (7·5-10·0)	9·7% (8·3-11·1)	10·4% (8·9-11·9)	11·1% (9·4-12·7)
PTSD, SUD, Crime history	34·0% (29·4-38·3)	37·7% (32·4-42·6)	40·2% (34·3-45·5)	44·0% (36·8-50·3)	45·3% (37·8-51·9)

Abbreviations: PTSD = post-traumatic stress syndrome, SUD = substance use disorder.

Table S6. Hazard ratios for sex-stratified models in the matched cohort

	N PTSD	(N TOTAL)	HR (95% CI)
**Stratified by sex (minimally-adjusted model)**			
Male	3 263	35 983	**7·3** (6·4 – 8·3)
Female	9 856	108 416	**7·6** (6·4 – 9·1)
**Stratified by sex (fully-adjusted model*)**			
Male	3 225	35 708	**6·4** (5·6 – 7·4)
Female	9 813	108 013	**6·4** (5·3 – 7·7)

Abbreviations: HR = Hazard Ratio, CI = confidence interval. Analyses include each matched set as separate strata. All models in this table are implicitly adjusted for sex, birth year, and county of residence in year of diagnosis. The minimally-adjusted model adjusts for sex, birth year, and county of residence in year of diagnosis through matching. The fully-adjusted model adjusts for parental characteristics and psychiatric comorbidities (major depressive disorder, schizophrenia, bipolar disorder, and attention deficit hyperactivity disorder/conduct disorder) diagnosed prior to PTSD diagnosis. For psychiatric comorbidities in individuals without PTSD, we used the matched diagnosed individual's (or diagnosed sibling's) date of PTSD diagnosis. *588 observations excluded due to missing information on parental characteristics.

Table S7. Descriptives for individuals suspected of violent crime in the matched cohort, as defined by the National Criminal Suspects Register

	**PTSD** ***(% total)***	**No PTSD** ***(% total)***
**History of violent crime suspicion**		
History of violent crime suspicion	2 206 *(16·8%)*	5 034 *(3·8%)*
**Violent crime suspicion during follow-up**		
Suspicion of violent crime during follow-up	1 611 *(12·3%)*	3 005 (*2·3%)*
Median age at suspicion in years (interquartile range)	31·0 (23·8 – 39·5)	30·8 (22·8 – 40·1)

Abbreviations: PTSD = post-traumatic stress disorder.

Table S8. Hazard ratios for stratified Cox regression using suspicion of violent crime as outcome

	**N PTSD**	**N TOTAL**	**Matched Cohort HR (95% CI)**
**Model 1** (minimally-adjusted model)	13 119	144 309	**6·1** (5·7 – 6·5)
**Model 2** (parental characteristics adjusted model)	13 038	143 721	**6·0** (5·6 – 6·3)
**Model 3** (fully-adjusted model: parental characteristics + prior psychiatric comorbidities)	13 038	143 721	**5·3** (4·9 – 5·7)
**Model 4** (fully-adjusted model; stratified by prior SUD)			
No prior SUD diagnosis	10 350	137 396	**5·0** (4·6 – 5·4)
Prior SUD diagnosis	2 688	6 325	**2·0** (1·7 – 2·4)
**Model 5** (fully-adjusted model including prior SUD; stratified by history of violent crime)			
No history of suspicion of violent crime	10 859	136 544	**4·3** (3·9 – 4·7)
History of suspicion of violent crime	2 179	7 177	**2·0** (1·7 – 2·3)

Abbreviations: HR = Hazard Ratio, CI = confidence interval, SUD = substance use disorder. Analyses include each matched set as separate strata in the matched cohort. All models in the matched cohort are implicitly adjusted for sex, birth year, and county of residence in year of diagnosis. The minimally-adjusted model (Model 1) adjusts for sex, birth year, and county of residence in year of diagnosis through matching. Model 2 additionally adjusts for parental characteristics, and Model 3 (the fully-adjusted model) adjusts for parental characteristics and psychiatric comorbidities (major depressive disorder, schizophrenia, bipolar disorder, and attention deficit hyperactivity disorder/conduct disorder) diagnosed prior to PTSD diagnosis. For psychiatric comorbidities in individuals without PTSD, we used the matched diagnosed individual's (or diagnosed sibling's) date of PTSD diagnosis. Models 4 and 5 are fully adjusted (adjusting for parental characteristics and psychiatric comorbidities) and stratified by prior SUD diagnosis and history of violent crime, respectively. Model 5 additionally includes adjustment for prior SUD.

Table S9. Hazard ratios for sensitivity analysis in the restricted birth matched cohort, including data for stratifying by SUD and history of violent crime

	**N PTSD**	**N TOTAL**	**HR (95% CI) (1958-)**	**N PTSD**	**N TOTAL**	**HR (95% CI) (1973-)**	**N PTSD**	**N TOTAL**	**HR (95% CI) (1983-)**
**Model 1** (minimally-adjusted model)	13 119	144 309	**7·4** (6·6 – 8·2)	8 180	89 980	**7·3** (6·4 – 8·3)	4 665	51 315	**6·6** (5·5 – 7·8)
**Model 2** (parental characteristics*)	13 038	143 721	**7·4** (6·6 – 8·2)	8 164	89 900	**7·2** (6·3 – 8·2)	4 658	51 273	**6·5** (5·5 – 7·8)
**Model 3** (fully-adjusted model: parental characteristics* + prior psychiatric comorbidities)	13 038	143 721	**6·4** (5·7 – 7·2)	8 164	89 900	**6·4** (5·5 – 7·4)	4 658	51 273	**6·3** (5·2 – 7·6)
**Model 4** (fully-adjusted model*; stratified by SUD by including interaction term)									
No SUD diagnosis	10 350	137 396	**6·1** (5·3 – 6·9)	6 406	89 597	**5·7** (4·8 – 6·8)	3 659	48 699	**5·6** (4·5 – 7·1)
SUD diagnosis	2 688	6 325	**1·9** (1·5 – 2·6)	1 758	4 303	**2·3** (1·6 – 3·2)	999	2 574	**2·3** (1·4 – 3·8)
**Model 5** (fully-adjusted model* including adjustment for prior SUD; stratified for history of violent crime by including interaction term)									
No history of violent crime	11 826	139 641	**5·0** (4·3 – 5·7)	7 443	87 628	**4·9** (4·1 – 6·0)	4 330	50 181	**4·9** (3·9 – 6·2)
History of violent crime	1 212	4 080	**2·2** (1·7 – 3·0)	721	2 272	**1·7** (1·2 – 2·5)	328	1 092	1·6 (0·9 – 2·8)

Abbreviations: HR = Hazard Ratio, CI = confidence interval, SUD = substance use disorder. Analyses include each matched set as separate strata. All models in this table are implicitly adjusted for sex, birth year, and county of residence in year of diagnosis. The minimally-adjusted model (Model 1) adjusts for sex, birth year, and county of residence in year of diagnosis through matching. Model 2 additionally adjusts for parental characteristics, and Model 3 (the fully-adjusted model) adjusts for parental characteristics and psychiatric comorbidities (major depressive disorder, schizophrenia, bipolar disorder, and attention deficit hyperactivity disorder/conduct disorder) diagnosed prior to PTSD diagnosis. For psychiatric comorbidities in individuals without PTSD, we used the matched diagnosed individual's (or diagnosed sibling's) date of PTSD diagnosis. Models 4 and 5 are fully adjusted (adjusting for parental characteristics and psychiatric comorbidities) and stratified by prior SUD diagnosis and history of violent crime, respectively. Model 5 additionally includes adjustment for prior SUD.

*Observations excluded due to missing information on parental characteristics: 588 (1958-); 80 (1973-); 42 (1983-)

Table S10. Hazard ratios for stratified Cox regression excluding individuals with psychiatric comorbidities preceding PTSD diagnosis

	N PTSD	N TOTAL	Matched Cohort HR (95% CI)	N PTSD	N TOTAL	Sibling Cohort HR (95% CI)
**Model 1** (minimally-adjusted model)	5 197	57 167	**5·8** (4·9 – 6·8)	4 167	10 286	**3·0** (2·2 – 4·2)
**Model 2** (fully-adjusted model: parental characteristics*)	5 172	56 905	**5·8** (4·9 – 6·9)	··	··	··
**Model 3** (fully-adjusted model*; stratified by history of violent crime)						
No history of violent crime	4 872	55 809	**5·4** (4·4 – 6·6)	3 934	9 768	**3·5** (2·4 – 5·1)
History of violent crime	300	1 096	**2·5** (1·5 – 4·1)	223	496	1·7 (0·8 – 3·7)

Abbreviations: HR = Hazard Ratio, CI = confidence interval, PTSD = post-traumatic stress disorder. Analyses include each matched set as separate strata in the matched cohort and each family as separate strata in the sibling cohort. All models in the matched cohort are implicitly adjusted for sex, birth year, and county of residence in year of diagnosis. All models in the sibling cohort are adjusted for sex and birth year. The minimally-adjusted model (Model 1) adjusts for sex, birth year, and county of residence in year of diagnosis through matching. Model 2 additionally adjusts for parental characteristics. Model 3 adjusts for parental characteristics and is stratified by history of violent crime. For siblings, parental characteristics only includes parental low income.

Psychiatric comorbidities we adjusted for in original analyses (excluded here): major depressive disorder (MDD), bipolar disorder, schizophrenia, attention deficit hyperactivity disorder (ADHD) and conduct disorder. *Excluded due to missing information on parental characteristics: 262 (matched cohort), 22 (sibling cohort)

Table S11. Hazard ratios for stratified Cox regression, separately for those diagnosed with PTSD prior to age 18 and those diagnosed age 18 or older in the matched cohort

	**N PTSD**	**N TOTAL**	**Aged less than 18**<br>**HR (95% CI)**	**N PTSD**	**N TOTAL**	**Aged 18 or above**<br>**HR (95% CI)**	**P-value for difference**
**Model 1** (minimally-adjusted model)	1 065	11 715	**6·2** (4·8 – 7·9)	12 054	132 594	**7·7** (6·9 – 8·6)	0.111
**Model 2** (parental characteristics adjusted model)	1 062	11 703	**6·1** (4·7 – 7·8)	11 976	132 018	**7·7** (6·8 – 8·6)	0.095
**Model 3**[a] (fully-adjusted model: parental characteristics + prior psychiatric comorbidities)	1 062	11 703	**6·1** (4·7 – 8·0)	11 976	132 018	**6·5** (5·7 – 7·4)	0.662

Abbreviations: PTSD = post-traumatic stress disorder, HR = Hazard Ratio, CI = confidence interval, SUD = substance use disorder. Analyses include each matched set as separate strata in the matched cohort. All models in the matched cohort are implicitly adjusted for sex, birth year, and county of residence in year of diagnosis.

The minimally-adjusted model (Model 1) adjusts for sex, birth year, and county of residence in year of diagnosis through matching. Model 2 additionally adjusts for parental characteristics, and Model 3 (the fully-adjusted model) adjusts for parental characteristics and psychiatric comorbidities diagnosed prior to PTSD diagnosis. For psychiatric comorbidities in individuals without PTSD, we used the matched diagnosed individual's (or diagnosed sibling's) date of PTSD diagnosis. Models 4 and 5 are fully adjusted (adjusting for parental characteristics and psychiatric comorbidities) and stratified by prior SUD diagnosis and history of violent crime, respectively. Model 5 additionally includes adjustment for prior SUD.

[a] No one aged less than 18 had had schizophrenia, so model was not adjusted for schizophrenia.

[b] Model not adjusted for conduct disorder because low number of exposed among those aged less than 18 with prior violent crime did not allow to fit model.

Figure S1. Matched and sibling cohorts, as obtained from the Swedish population

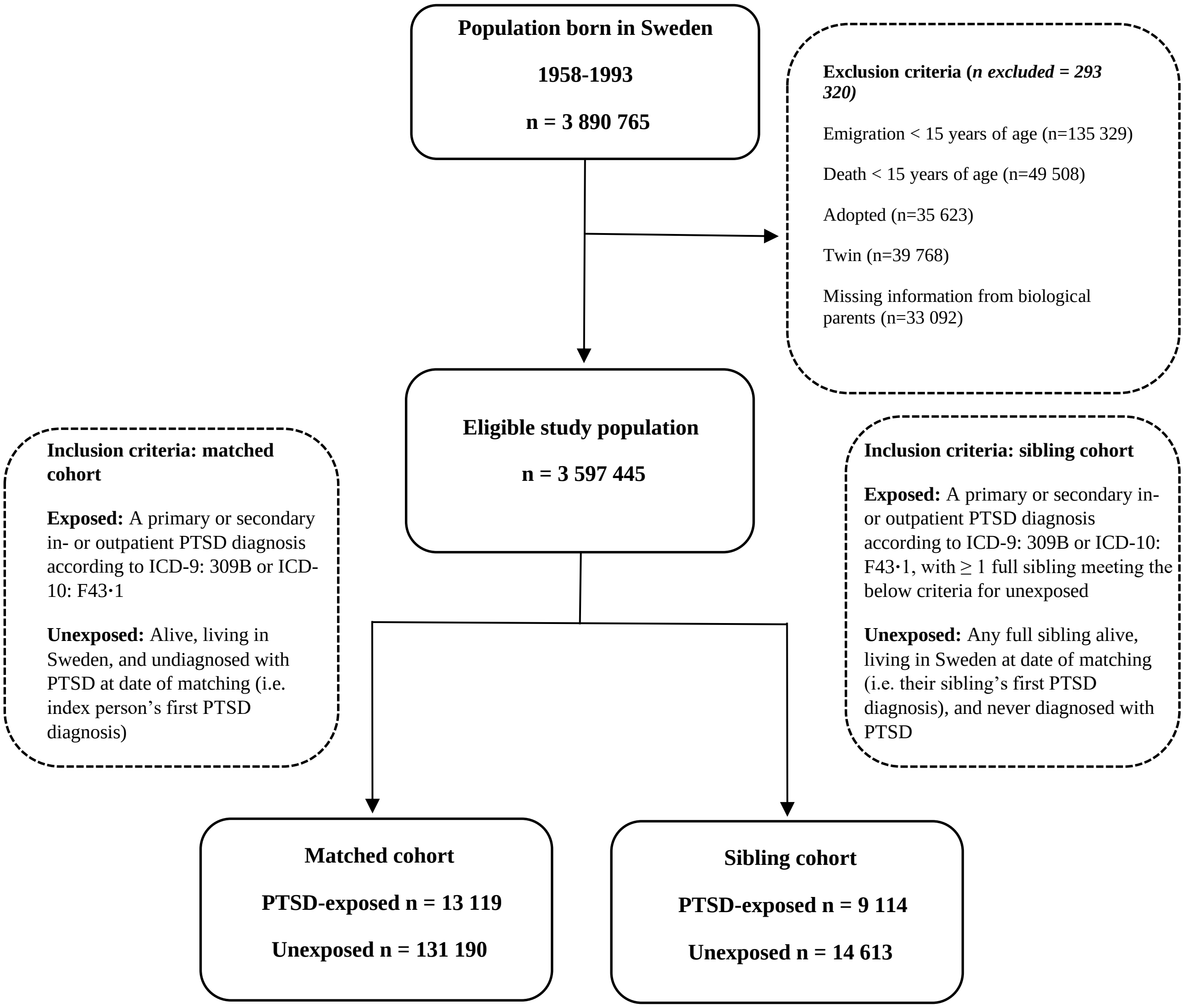

Abbreviations: PTSD = post-traumatic stress disorder, ICD = International Classification of Diseases.

Figure S2. Assessment of non-proportional hazards of violent crime over follow-up time, comparing individuals with PTSD with matched controls in a stratified Cox regression (stratified on matching cluster). Estimates with 95% confidence intervals and Schoenfeld residuals.

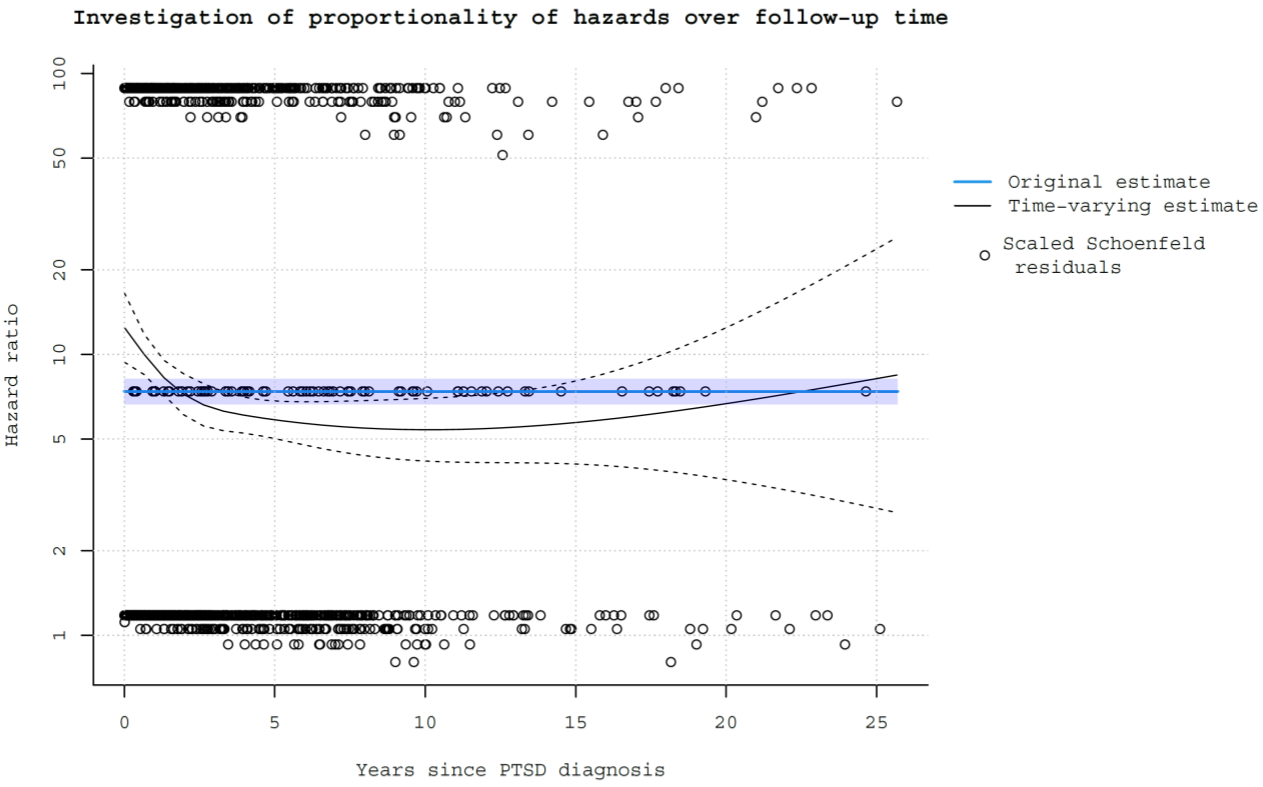

Abbreviations: PTSD = post-traumatic stress disorder. Note: Because of stratified analysis, the Schoenfeld residuals have a pattern with relatively discrete possible values and may be hard to interpret. Given sample size and shape of time-varying hazard ratio over time, the proportionality assumption was deemed acceptable.
